# The associations of dental aesthetics, oral health-related quality of life and satisfaction with aesthetics in an adult population

**DOI:** 10.1093/ejo/cjac075

**Published:** 2023-01-23

**Authors:** Linnea Närhi, Minttu Mattila, Mimmi Tolvanen, Pertti Pirttiniemi, Anna-Sofia Silvola

**Affiliations:** Department of Oral Development and Orthodontics, Oral Health Sciences, Faculty of Medicine, University of Oulu, Finland; Medical Research Center Oulu (MRC Oulu), Oulu University Hospital, Finland; Department of Oral Development and Orthodontics, Oral Health Sciences, Faculty of Medicine, University of Oulu, Finland; Faculty of Medicine, University of Oulu, Finland; Department of Oral Development and Orthodontics, Oral Health Sciences, Faculty of Medicine, University of Oulu, Finland; Medical Research Center Oulu (MRC Oulu), Oulu University Hospital, Finland; Department of Oral Development and Orthodontics, Oral Health Sciences, Faculty of Medicine, University of Oulu, Finland; Medical Research Center Oulu (MRC Oulu), Oulu University Hospital, Finland

## Abstract

**Aim:**

The aim of this study was to investigate the gender-specific associations between dental aesthetics, oral health-related quality of life (OHRQoL), and satisfaction with dental aesthetics in an adult population.

**Materials and methods:**

The study population consisted of 1780 individuals (822 males and 958 females) from the Northern Finland Birth Cohort 1966 (NFBC1966). Dental aesthetics were evaluated from digital 3D dental models using the Aesthetic Component (AC) of the Index of Orthodontic Treatment Need (IOTN). Layperson and orthodontist panels evaluated the dental aesthetics of a smaller sample (*n* = 100). OHRQoL was measured using the Oral Health Impact Profile (OHIP-14) questionnaire. Satisfaction with dental aesthetics was asked with one separate question. Gender-specific analyses consisted of Mann–Whitney *U*-tests and Spearman’s correlation coefficients.

**Results:**

More than half of the population had an aesthetically acceptable occlusion, and most of the individuals were satisfied with the aesthetics. The most severe aesthetic impairments were associated with the psychological dimensions of OHIP-14. There were significant but weak associations of AC and satisfaction with aesthetics, and satisfaction with aesthetics and OHRQoL. Significant gender differences were found, men having higher mean AC scores but women reporting lower OHRQoL.

**Conclusion:**

At the population level, most of the individuals were satisfied with their aesthetics, despite different dental aesthetic conditions. The most severe aesthetic impairments were associated with decreased psychological well-being, women reporting more impacts compared to men.

## Introduction

Malocclusion can be defined as different misalignments of the teeth and/or jaws. These misalignments can affect occlusal function but may also significantly impair an individual’s dentofacial aesthetics (1–3). The appearance of teeth and smile affect the attractiveness of the face, which has an important role in social interaction (2–4). Dentofacial aesthetics have been found to have a significant effect on how people perceive themselves and how they are perceived by others (2–5). Judgements based on dentofacial aesthetics have been found to extend not only to the attractiveness of others but also to their personality and psychological and intellectual qualities (2, 3), whereas favourable smile aesthetics have been considered to provide social benefits (6, 7).

The social acceptance of dental aesthetic impairments may affect the satisfaction and OHRQoL of the individual. Concerns about dental aesthetics are one of the most common reasons for dental dissatisfaction (5), being also the main reason to seek orthodontic treatment (8). The impacts of malocclusions on daily lives can be evaluated by investigating the associations with oral health-related quality of life (OHRQoL), meaning the physical, psychological and social well-being of the individual (9, 10). Normatively assessed malocclusion has generally been found to associate with OHRQoL (11). The OHRQoL impacts depend on both the aesthetic and functional aspects of occlusion, although patients seem to be more concerned about aesthetics (5). Dental aesthetic impairments have been found to have a negative impact on OHRQoL, especially on its psychological, social and emotional dimensions (12–16); however, opposite findings have also been reported (17).

The evaluation of dental aesthetics is complex and affected by culture and social environment and the measures used (18–20), and it may also differ between patients, laypersons, and dental professionals (15, 20–22). Dental professionals use standardized methods or measures when evaluating dental appearance and orthodontic treatment need (18, 23). However, an individual’s own perception and satisfaction with his/her dental aesthetics may be inconsistent with normatively assessed malocclusion, some people being very concerned about minor irregularities while others are not disturbed by even severe aesthetical issues (8, 14, 24).

In the associations between malocclusions and dental aesthetics and OHRQoL, different findings have been reported, depending on the study population and study design (11). Age and gender have been found to be important variables influencing these associations, as well (11, 12, 24). Most of the previous population-based studies were conducted among children or adolescents (11, 12), whereas scientific evidence on the associations of dental aesthetics and OHRQoL impacts in an adult population is still limited. The aim of this study was to investigate the gender-specific associations between dental aesthetics, OHRQoL, and satisfaction with dental aesthetics in an adult population.

## Materials and methods

**Figure 1:**
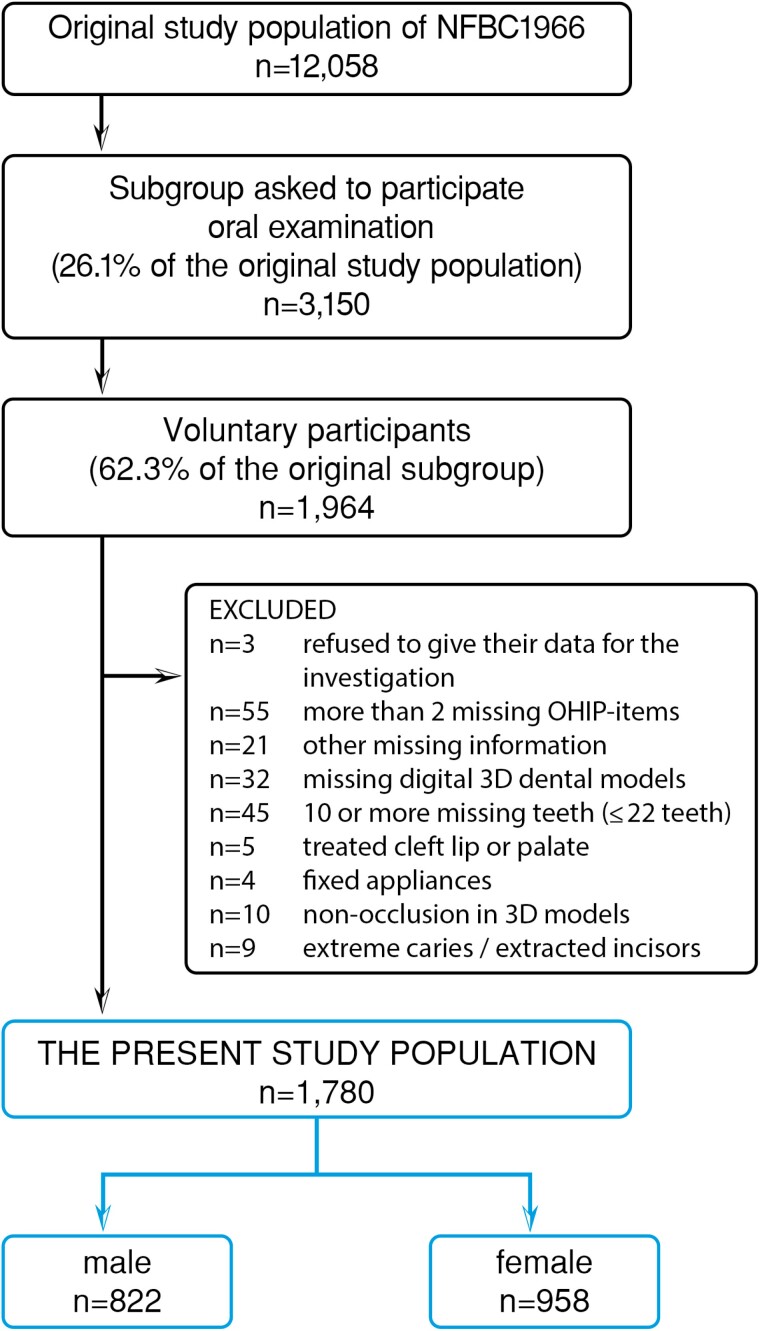
The flow chart of the study population.

This study was executed as part of the Northern Finland Birth Cohort 1966 (NFBC1966) study, which originally consisted of all children whose expected time of delivery was in 1966 in the two northernmost provinces of Finland (*n* = 12 058) (25, 26). In connection with the 46-year follow-up survey (2012–2013), a subgroup of 3150 subjects was asked to participate in an oral examination, and a total of 1964 subjects (62.3%) (912 males and 1052 females) decided to participate (26). The final study population (*n* = 1780) consisted of 822 males and 958 females. Exclusion criteria were the following: refusing to give data for the investigation (*n* = 3), missing digital 3D dental models or other missing information (*n* = 108), 10 or more missing teeth (*n* = 45), cleft lip or palate (*n* = 5), fixed appliances (*n* = 4), non-occlusion in 3D models (*n* = 10), extreme caries or extracted incisors (*n* = 9). The flow chart of the study population is shown in Figure 1.

In connection with the oral examination, digital 3D dental models were obtained using an iTero 3D scanner (Cadent, San Jose, CA, USA). The occlusion was registered in inter-cuspal position. 3Shape Ortho Analyzer^TM^ software (Copenhagen, Denmark) was used in analysing occlusal aesthetics from the digital 3D models.

Dental aesthetics were evaluated from the frontal view of the digital 3D dental models, using the Aesthetic Component (AC) of the Index of Orthodontic Treatment Need (IOTN). The AC of the IOTN is a commonly used instrument for evaluation of dental aesthetics. It includes ten separate colour photographs which can be graded on a scale from 1 to 10 according to dental attractiveness, 1 meaning the most attractive and 10 the least attractive dental appearance (23, 27). The aesthetic evaluations with the AC were made by one calibrated dentist (L.N.).

**Figure 2:**
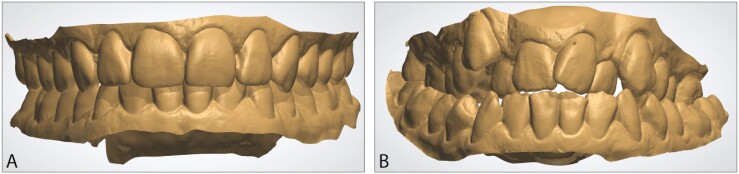
The reference pictures selected to indicate the extremities of dental aesthetics in the study population, (A) presenting the most attractive, and (B) the least attractive dental appearance.

In addition, orthodontist and layperson panels consisting of five orthodontists (2 men, 3 women), and ten laypersons (5 men, 5 women), were organized to evaluate the dental aesthetics. The orthodontists were staff working at University of Oulu, and the laypersons were people with no dental education. For the panel evaluations, a randomly selected representative sample (*n* = 100) of the 3D dental models was collected. The panel groups evaluated the dental aesthetics from the frontal view of the 3D dental models using a scale from 1 to 10, consistently with the AC of the IOTN. The panel groups had the reference pictures for the most and the least attractive dental appearances (Figure 2), and were asked to evaluate just the occlusion.

The participants of this study completed standardized questionnaires including questions on orthodontic treatment history (0 = no, 1 = yes), OHRQoL and satisfaction with dental aesthetics. To investigate OHRQoL, Finnish translation of the Oral Health Impact Profile-14 (OHIP-14) questionnaire was used (28, 29). OHIP-14 consists of 14 questions, two questions for each of the seven conceptual dimensions of OHRQoL. The questions investigate how often the individual has experienced functional limitation, physical pain, psychological discomfort, physical disability, psychological disability, social disability and handicap. Each question was answered on a five-point Likert-type scale (0 = never, 1 = hardly ever, 3 = fairly often, 4 = very often) concerning the previous month. Participants’ satisfaction with their dental appearance was asked with a separate question ‘how satisfied are you with your dental aesthetics’, and answered on a Likert-type scale (1 = very satisfied, 2 = quite satisfied, 3 = quite unsatisfied, 4 = very unsatisfied).

The participation in this study was voluntary, and the participants had the right to decline from giving their data or withdraw from the study at any time. All the participants signed a written informed consent form. The Ethical Committee of the Northern Ostrobothnia Hospital District approved the study protocol (74/2011).

### Statistical analysis

Due to asymmetric distributions, both mean and median values of AC, OHIP severity and dimensions and satisfaction with dental aesthetics were calculated, and Mann–Whitney *U*-test was used to examine the differences. For further analyses, AC was trichotomized into AC 1–4 (aesthetically acceptable occlusion), AC 5–7 (moderately impaired aesthetics) and AC 8–10 (severely impaired aesthetics). The AC 1–4 group (aesthetically acceptable occlusion) and the satisfaction with dental aesthetics 1 group (very satisfied) were selected as reference groups. The mean OHIP severity and dimension scores and satisfaction with dental aesthetic scores of AC 5–7 and AC 8–10 groups were compared to those of the AC 1–4 group. The mean OHIP severity scores of the satisfaction with dental aesthetic groups 2 (quite satisfied), 3 (quite unsatisfied) and 4 (very unsatisfied) were compared separately to the score of group 1 (very satisfied). To examine the differences between groups, Mann–Whitney *U*-test was used, and the analyses were conducted separately for both genders. Spearman’s rho correlation coefficient was used to assess the relationship between AC and satisfaction with aesthetics, and between satisfaction with aesthetics and the OHIP severity. The same analyses were used to assess the associations between the panel evaluations and the mean OHIP severity and dimension scores and satisfaction with aesthetic scores, as well as the correlation between the panel evaluations and satisfaction with aesthetics. The level of statistical significance was set to *P*-value of < 0.05. Statistical analyses were conducted using IBM SPSS Statistics 27.0 (SPSS Inc., Chicago, IL, USA).

## Results

The mean OHIP-14 severity score in the study population was 3.60. In the total study population, AC 1–4 (aesthetically acceptable occlusion) was registered for 47.4% of men and 54.7% of women, AC 5–7 (moderately impaired aesthetics) for 45.1% of men and 39.7% of women, and AC 8–10 (severely impaired aesthetics) for 7.4% of men and 5.6% of women. The majority of the study population (77.9% of men and 76.0% of women) reported being very satisfied or quite satisfied with their dental aesthetics. No statistically significant association of AC and overall OHRQoL was found, but satisfaction with dental aesthetics was associated with OHIP severity in both genders (*P* < 0.05) (Table 1). No difference in the AC scores, OHRQoL or satisfaction with dental aesthetics was found between subjects with and without orthodontic treatment history.

**Table 1.** Characteristics of the study population and mean and median values of the Oral Health Impact Profile (OHIP-14) severity score.

	Male				Female			
	OHIP severity				OHIP severity			
	*n* (%)	Mean	Md	*P*	*n* (%)	Mean	Md	*P*
ALL (*n* = 1780)	822	3.14	2		958	3.99	2	
Self-reported orthodontic treatment history								
Yes	137 (16.7)	2.64	1	0.074	193 (20.1)	3.95	2	0.592
No	685 (83.3)	3.24	2		765 (79.9)	4.00	2	
Satisfaction with aesthetics								
1 (reference group)	64 (7.8)	1.09	0		73 (7.6)	2.14	1	
2	576 (70.1)	2.37	1	0.001^a^^,^^*^	655 (68.4)	2.95	2	0.063^a^
3	168 (20.4)	5.69	4	<0.001^a^^,^^*^	185 (19.3)	6.31	5	<0.001^a^^,^^*^
4	14 (1.7)	13.57	10	<0.001^a^^,^^*^	45 (4.7)	12.60	10	<0.001^a^^,^^*^
Aesthetic component of the IOTN								
1–4 (reference group)	390 (47.4)	3.06	1		524 (54.7)	3.93	2	
5–7	371 (45.1)	3.09	2	0.951^b^	380 (39.7)	3.86	2	0.444^b^
8–10	61 (7.4)	3.93	2	0.110^b^	54 (5.6)	5.44	3.5	0.184^b^

Mann–Whitney *U*-test.

^a^Satisfaction with dental aesthetics groups 2 (quite satisfied), 3 (quite unsatisfied) and 4 (very unsatisfied) compared separately to the group 1 (very satisfied).

^b^AC 5–7 (moderately impaired aesthetics) and AC 8–10 (severely impaired aesthetics) compared separately to AC 1–4 (aesthetically acceptable occlusion).

^*^ *P* < 0.05.

Some significant differences between genders were found. The mean AC score differed significantly between genders, with males having slightly higher mean AC score compared to females (*P* < 0.001). The mean OHIP severity score was 3.14 in men and 3.99 in women (*P* = 0.001), and women reported more psychological discomfort, physical disability, and psychological disability compared to men (*P* < 0.001, *P* = 0.021, *P* < 0.001). No difference between genders was found in satisfaction with dental aesthetics (Table 2).

**Table 2.** The gender-specific mean and median (*Q*_1_−*Q*_3_) values of the Aesthetic Component (AC) of the Index of Orthodontic Treatment Need (IOTN), the Oral Health Impact Profile (OHIP-14) severity and dimensions, and satisfaction with dental aesthetics.

	MALE *n* = 822		FEMALE *n* = 958		*P*
	Mean	Md (*Q*_1_−*Q*_3_)	Mean	Md (*Q*_1_−*Q*_3_)	
AC	4.91	5 (4–6)	4.55	4 (3–6)	<0.001^*^
OHIP severity	3.14	2 (0–4)	3.99	2 (0–6)	0.001^*^
Functional limitation	0.17	0 (0–0)	0.20	0 (0–0)	0.887
Physical pain	1.33	1 (0–2)	1.47	1 (0–2)	0.481
Psychological discomfort	0.68	0 (0–1)	0.98	0 (0–2)	<0.001^*^
Physical disability	0.19	0 (0–0)	0.28	0 (0–0)	0.021^*^
Psychological disability	0.36	0 (0–0)	0.55	0 (0–1)	<0.001^*^
Social disability	0.19	0 (0–0)	0.21	0 (0–0)	0.552
Handicap	0.22	0 (0–0)	0.29	0 (0–0)	0.052
Satisfaction with aesthetics	2.16	2 (2–2)	2.21	2 (2–2)	0.266

Mann–Whitney *U*-test. *P*-values for the difference between genders.

^*^ *P* < 0.05.

Table 3 shows the associations of the AC and the mean OHIP severity and dimension scores. In women, severely impaired dental aesthetics (AC 8–10) was associated with psychological discomfort, psychological disability, and handicap (*P* = 0.001, *P* = 0.022, *P* < 0.001, respectively), while in men, only an association with psychological disability was found (*P* = 0.010). Impaired dental aesthetics was associated with satisfaction with dental aesthetics in both genders (*P* < 0.001) (Table 3).

**Table 3.** The gender-specific mean and median values of the Oral Health Impact Profile (OHIP-14) severity and dimensions, and satisfaction with dental aesthetics for different dental aesthetic groups.

	AC 1–4		AC 5–7		*P*	AC 8–10		*P*
	Mean	Md	Mean	Md		Mean	Md	
MALE	*n* = 390		*n* = 371			*n* = 61		
OHIP severity	3.06	1	3.09	2	0.951	3.93	2	0.110
Functional limitation	0.15	0	0.18	0	0.613	0.18	0	0.536
Physical pain	1.34	1	1.28	1	0.463	1.62	1	0.362
Psychological discomfort	0.64	0	0.70	0	0.302	0.85	0	0.156
Physical disability	0.20	0	0.18	0	0.449	0.18	0	0.943
Psychological disability	0.33	0	0.36	0	0.546	0.54	0	0.010^*^
Social disability	0.18	0	0.19	0	0.732	0.18	0	0.350
Handicap	0.22	0	0.20	0	0.759	0.38	0	0.087
Satisfaction with dental aesthetics	2.02	2	2.25	2	<0.001^*^	2.52	2	<0.001^*^
FEMALE	*n* = 524		*n* = 380			*n* = 54		
OHIP severity	3.93	2	3.86	2	0.444	5.44	3.5	0.184
Functional limitation	0.18	0	0.21	0	0.189	0.30	0	0.245
Physical pain	1.54	1	1.41	1	0.607	1.33	1	0.438
Psychological discomfort	0.91	0	0.97	0	0.341	1.65	1	0.001^*^
Physical disability	0.33	0	0.23	0	0.196	0.22	0	0.211
Psychological disability	0.51	0	0.55	0	0.518	0.91	0	0.022^*^
Social disability	0.20	0	0.21	0	0.828	0.37	0	0.552
Handicap	0.26	0	0.29	0	0.230	0.67	0	<0.001^*^
Satisfaction with dental aesthetics	2.08	2	2.32	2	<0.001^*^	2.74	3	<0.001^*^

AC 5–7 (moderately impaired aesthetics) and AC 8–10 (severely impaired aesthetics) were compared separately to AC 1–4 (aesthetically acceptable occlusion). Mann–Whitney *U*-test.

^*^ *P* < 0.05.

**Figure 3:**
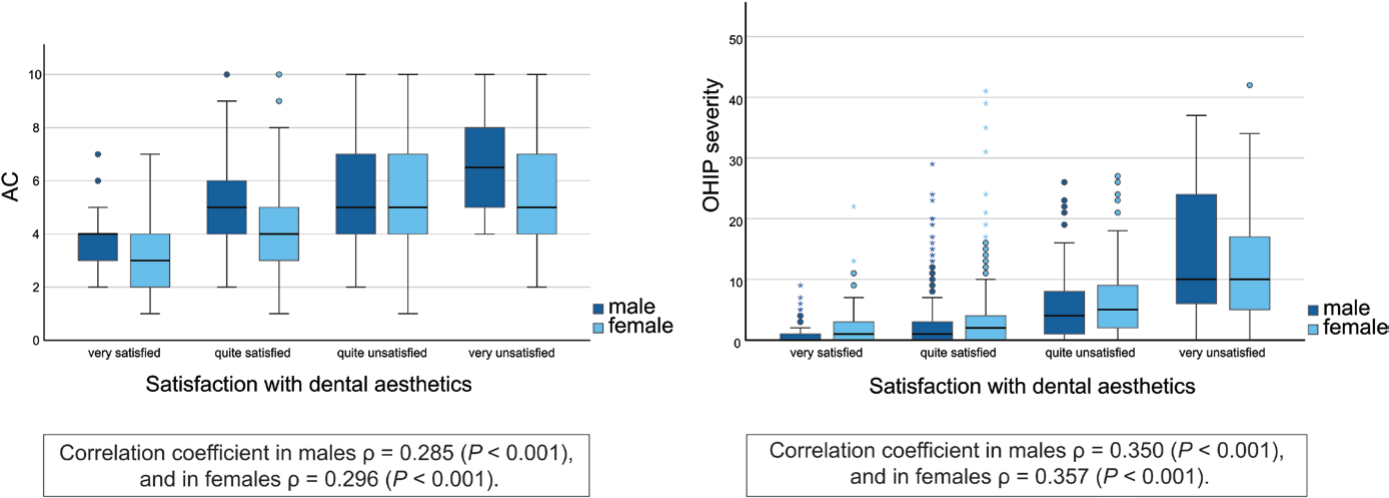
Boxplots presenting median values and ranges of the Aesthetic Component (AC) and the Oral Health Impact Profile (OHIP-14) severity in different satisfaction with dental aesthetic groups (*n* = 1780). Spearman’s rho correlation coefficient.

When both AC and satisfaction with dental aesthetics were continuous variables, the correlation coefficient was *ρ* = 0.285 (*P* < 0.001) in males and *ρ* = 0.296 (*P* < 0.001) in females. Those who were more dissatisfied with their dental aesthetics had also lower OHRQoL. Correlation coefficient for satisfaction with dental aesthetics and OHIP severity was *ρ* = 0.350 (*P* < 0.001) in males and *ρ* = 0.357 (*P* < 0.001) in females (Figure 3).

**Figure 4:**
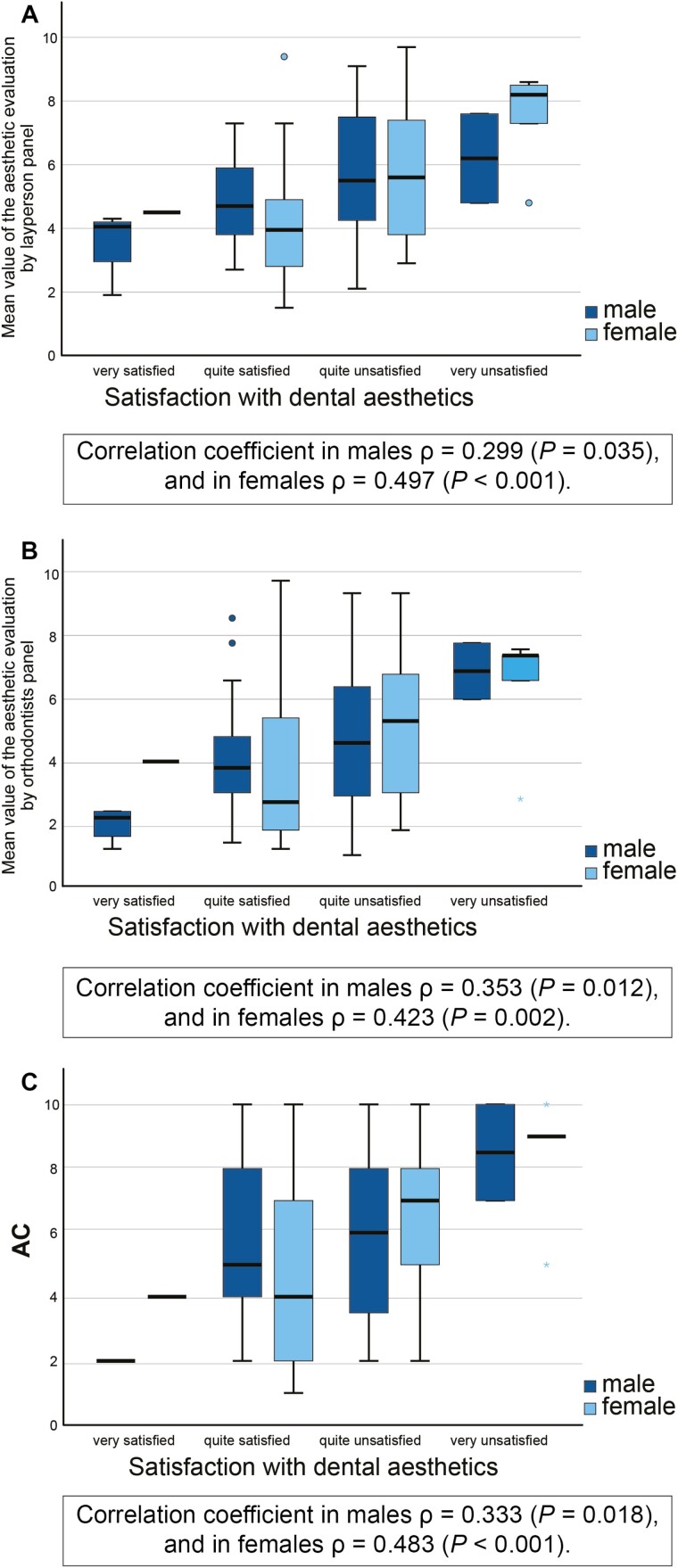
Boxplots presenting median values and ranges of aesthetic evaluations of layperson (A) and orthodontist (B) panels and the AC (C) in different satisfaction with dental aesthetic groups (*n* = 100). Spearman’s rho correlation coefficient.

In layperson panel evaluations, a statistically significant association of severely impaired aesthetics was found with OHIP severity and psychological disability in men (*P* = 0.049, *P* = 0.012, respectively), and with handicap in women (*P* = 0.022), and satisfaction with dental aesthetics in both genders (*P* = 0.002 in men, *P* = 0.005 in women). In orthodontist panel evaluations, only associations of impaired aesthetics and handicap and satisfaction with dental aesthetics in women (*P* = 0.030, *P* = 0.007, respectively) were found. Correlation coefficient for layperson panel aesthetic evaluation and satisfaction with dental aesthetics was *ρ* = 0.299 (*P* = 0.035) in males and *ρ* = 0.497 (*P* < 0.001) in females; for orthodontist panel aesthetic evaluation and satisfaction with aesthetics, correlation coefficient was *ρ* = 0.353 (*P* = 0.012) in males and *ρ* = 0.423 (*P* = 0.002) in females (Figure 4).

## Discussion

This study assessed the associations between dental aesthetics, OHRQoL and satisfaction with dental aesthetics in a Finnish adult population. Severe aesthetic impairments had a negative effect on psychological quality of life, but most of the individuals were satisfied with their aesthetics despite different levels of aesthetic impairments.

The prevalence of aesthetic impairments measured by AC of the IOTN varies in different study populations in previous studies (16, 18, 30). In this study, more than half of the population were evaluated to have AC 1– 4, considered as aesthetically acceptable occlusion, and severe aesthetic impairments (AC 8–10) were registered for only 6.5% of the study population.

The grading of dental aesthetics has been considered rather subjective due to individual variation in the acceptance of dental features (2). According to previous studies, laypersons’ perceptions of dental aesthetics may differ from professionals’ assessment (15, 20–22). However, in this study, the aesthetic evaluations of layperson and orthodontist panels were parallel to the AC evaluations, and their associations with OHRQoL and satisfaction with aesthetics did not remarkably differ from those of AC.

Dental aesthetics are of great value in social interaction. Previous studies have emphasized the importance of an attractive smile and dental appearance on the first impression of another person and on social acceptance (4, 31). Persons with normal occlusion and favourable dentofacial aesthetics have been considered as more intelligent and attractive, to have a more favourable personality, and to be more successful (2, 3, 6, 7). In children, impaired dental appearance is the most common reason for bullying (32). At the individual level, social judgment and the significance of dental aesthetics may become apparent as psychosocial OHRQoL impacts and dissatisfaction with dental aesthetics.

In the present study population, one third of the individuals did not report any OHRQoL impacts, and therefore, the mean OHIP-14 severity score was low. More than 75% of the individuals were satisfied with their dental aesthetics, which was in line with previous population-based studies (5, 30, 33). The percentage of severe aesthetic impairments was slightly higher among men, but both genders were equally satisfied with their dental aesthetics. In general, females tend to be more critical of their dental appearance and more sensitive to OHRQoL impacts compared to males, and to consider well-aligned teeth more important (17, 30, 34). In parallel to previous studies, women reported slightly more OHRQoL impacts compared to men (12, 24).

In this study, dental aesthetics was not statistically significantly associated with overall OHIP-14. Previous studies have reported stronger association between dental aesthetics and OHRQoL. In orthodontic patients, severely compromised dental aesthetics have been found to be clearly associated with OHRQoL (15, 24). In young adults, even minor dental aesthetic impairments have been reported to have significant OHRQoL impacts (13), and impacts have been observed in most of the OHIP-14 scale values (16). However, most of the studies suggest that the effect of dental aesthetic impairments can mostly be seen in the psychosocial dimensions of OHRQoL (12, 14, 35). This was noticed in this study as well, as only the most severely impaired dental aesthetics increased psychological discomfort, psychological disability and handicap in women, and psychological disability in men.

Self-perception and satisfaction with dental appearance has been considered to be related to the severity of irregularities (5, 16). In this study, a statistically significant, albeit weak, correlation was found between normatively measured dental aesthetics and satisfaction with aesthetics. Interestingly, major individual variation was seen. There were individuals whose normatively assessed aesthetics could be considered optimal, but who were nevertheless quite or very unsatisfied with their aesthetics. In contrast, some individuals reported to be quite satisfied with their dental aesthetics despite severe aesthetic impairments. Similar findings have been reported among Nigerian and Brazilian adolescents (19, 24).

There was also a weak but statistically significant association between satisfaction with dental aesthetics and OHRQoL. Those who were more unsatisfied had higher OHIP severity scores, meaning lower OHRQoL, and this was seen in both genders. Orthodontic patients who are unsatisfied with their dental aesthetics have been found to be more likely to report oral impacts than those who are more satisfied (15). This might reflect their reasons to seek orthodontic treatment as the psychosocial impact of dental aesthetics has a strong influence on the decision to seek treatment (8, 24).

Orthodontic treatment has previously been found to significantly improve aesthetical appearance and satisfaction with dental appearance as well as psychological OHRQoL (15, 34). In a previous study in young Finnish adults, the odds for being satisfied were almost three times higher in treated subjects (33). However, the satisfaction levels may depend on the long-term result of the orthodontic treatment and the level of orthodontic treatment need in the untreated group (34). In the present study, dental aesthetics, satisfaction with dental aesthetics and OHRQoL did not differ between those with and without orthodontic treatment history. Only 18.5% of this study population had received any kind of orthodontic treatment, and different levels of aesthetic impairments still exist among the treated individuals due to insufficient treatment or relapse, which may explain the similar levels of satisfaction and OHRQoL. A previous study in this study population found the orthodontic treatment history to have a positive impact on OHRQoL, although it was only seen in the multivariate model, and therefore the impact should be considered limited (36).

The strength of this study is a nationally representative study population, with individuals of the same age and from the same region. The participation rate was high. The methods used to assess dental aesthetics and OHRQoL in this study are widely accepted and used (11, 35, 37, 38). In panel evaluations, a modified protocol was used, and only the references for the most and the least attractive dental appearance were shown.

The main limitation of this study is the chosen OHRQoL measure. OHIP-14 has not been designed to detect OHRQoL impacts related to orthodontic problems, and some of the questions are not relevant to dental aesthetic impacts (37). Also, the OHIP-14 has been shown not to work properly in population-based studies (39). This may partly explain the weak association of dental aesthetics, satisfaction with aesthetics and OHRQoL in this study.

In this study, no mathematical correction was made for multiple comparisons, since there were only three main components: satisfaction, aesthetic and OHIP. The statistical analysis resulted in relatively many significant *P*-values, indicating support to the study hypotheses.

In addition to malocclusion, there are many other oral/dental factors influencing the dentofacial aesthetics (5, 22, 31). Especially, the colour of the teeth has been found to be an important part of smile aesthetics and one of the main reasons for dental dissatisfaction (31). The digital 3D dental models used in this study did not show colours; therefore, the colour of the teeth did not affect the aesthetic evaluation. The evaluation panel members were also advised not to pay attention to other dental features than occlusion. Although the exclusion criteria in this study included other oral health conditions markedly affecting dental appearance, different dental/oral health factors may have confounded the results (29).

The OHRQoL impacts and satisfaction with aesthetics reflect the individual’s subjective perception. Therefore, it is obvious that different individual characteristics such as age, gender, sociodemographic factors, experiences and expectations, and personality have a great impact on these perceptions (10, 14, 17, 24). It has even been suggested that high aesthetic concern and psychological characteristics of the individual are better predictors of OHRQoL impacts than dental features themselves (40). The impact of these subjective factors may explain the major individual variation in the findings of this study.

In general, younger people have been considered to be more concerned with their dental aesthetics, which reflects the value of aesthetic appearance at younger age (14). This study corroborates the finding that most of the individuals seem to adapt to their condition and to be satisfied with their dental aesthetics in mature adulthood (41).

Satisfaction with aesthetics and OHRQoL are influenced by environmental/cultural norms, including aesthetic acceptability and perception and sociocultural importance of dental aesthetics, as well as treatment availability (5, 11, 17, 19, 20). A recent study comparing Finnish and Brazilian adult populations confirmed the view that cultural factors influence the psychosocial impacts of dental aesthetics (42). Therefore, the results of this study should be generalized with caution.

This population-based study shows that although dental aesthetic impairments seem to associate with satisfaction and psychosocial impacts, there are clearly some differences in individual perception and experience. Overall, the results of this study underline the view that the patient’s perception of his/her dental appearance should be emphasized when planning orthodontic treatment (16, 43).

## Conclusion

- More than half of the individuals had aesthetically acceptable occlusion.
- Most of the study population were satisfied and had no or little OHRQoL impacts despite different levels of dental aesthetic impairments.
- The most severe aesthetic impairments were associated with the psychological dimensions of OHIP-14, women reporting more impacts compared to men.
- Major individual variation was found in the associations between dental aesthetics, OHRQoL and satisfaction with aesthetics.

## Data Availability

NFBC data is available from the University of Oulu, Infrastructure for Population Studies. Permission to use the data can be applied for research purposes via the electronic material request portal. In the use of data, we follow the EU General Data Protection Regulation (679/2016) and the Finnish Data Protection Act. The use of personal data is based on cohort participants’ written informed consent at their latest follow-up examination, which may cause limitations to its use. For more information, please contact the NFBC project centre (NFBCprojectcenter@oulu.fi) and visit the cohort website.
